# Childhood Exposure to Phthalates: Associations with Thyroid Function, Insulin-like Growth Factor I, and Growth

**DOI:** 10.1289/ehp.0901331

**Published:** 2010-07-09

**Authors:** Malene Boas, Hanne Frederiksen, Ulla Feldt-Rasmussen, Niels E. Skakkebæk, Laszlo Hegedüs, Linda Hilsted, Anders Juul, Katharina M. Main

**Affiliations:** 1 Department of Growth and Reproduction and; 2 Department of Medical Endocrinology, University Hospital of Copenhagen, Rigshospitalet, Copenhagen, Denmark; 3 Department of Endocrinology and Metabolism, Odense University Hospital, Odense, Denmark; 4 Department of Clinical Biochemistry, University Hospital of Copenhagen, Rigshospitalet, Copenhagen, Denmark

**Keywords:** growth, insulin-like growth factor I, phthalate, thyroid

## Abstract

**Background:**

Phthalates are widely used chemicals, and human exposure is extensive. Recent studies have indicated that phthalates may have thyroid-disrupting properties.

**Objective:**

We aimed to assess concentrations of phthalate metabolites in urine samples from Danish children and to investigate the associations with thyroid function, insulin-like growth factor I (IGF-I), and growth.

**Methods:**

In 845 children 4–9 years of age, we determined urinary concentrations of 12 phthalate metabolites and serum levels of thyroid-stimulating hormone, thyroid hormones, and IGF-I.

**Results:**

Phthalate metabolites were detected in all urine samples, of which monobutyl phthalate was present in highest concentration. Phthalate metabolites were negatively associated with serum levels of free and total triiodothyronine, although statistically significant primarily in girls. Metabolites of di(2-ethylhexyl) phthalate and diisononyl phthalate were negatively associated with IGF-I in boys. Most phthalate metabolites were negatively associated with height, weight, body surface, and height gain in both sexes.

**Conclusions:**

Our study showed negative associations between urinary phthalate concentrations and thyroid hormones, IGF-I, and growth in children. Although our study was not designed to reveal the mechanism of action, the overall coherent negative associations between urine phthalate and thyroid and growth parameters may suggest causative negative roles of phthalate exposures for child health.

A normal thyroid function is important for growth and neurological development in children, and hypothyroidism in childhood is accompanied by growth retardation. A growing number of reports have indicated that environmental chemicals can interfere with thyroid function [reviewed by Boas et al. (2006)]. Both experimental and observational studies of wildlife and humans have suggested specific chemicals to have thyroid-disrupting properties, including recent investigations of phthalates. Phthalates are widely used industrial chemicals that are applied in a large variety of commercial products—for example, as plasticizers in toys, personal care products, and building materials, including wall paint (Sathyanarayana et al. 2008; Schettler 2006; Wormuth et al. 2006). Thus, human exposure is extensive, as demonstrated in numerous studies by quantifying phthalate metabolites in urine samples from adults and children (Becker et al. 2004; Meeker et al. 2007; Silva et al. 2004). Most human studies have investigated reproductive effects of phthalate exposure, but recent studies also indicated thyroid-disrupting effects. Thus, serum levels of free thyroxine (T_4_) and total triiodothyronine (T_3_) in adult men were negatively associated with concentrations of metabolites of di(2-ethylhexyl) phthalate (DEHP) (Meeker et al. 2007). In pregnant women urinary concentrations of metabolites of di-*n*-butyl phthalate (DBP) were negatively correlated with serum levels of free and total T_4_ (Huang et al. 2007).

The mechanisms of thyroid-disruption may be multiple, because experimental studies have suggested that phthalates interfere with binding of T_3_ to transport proteins (Ishihara et al. 2003), interacting with the active T_3_ uptake at the plasma membrane (Shimada and Yamauchi 2004) or exerting antagonistic activity at the thyroid receptors (Shen et al. 2009).

Phthalate exposure in children is probably higher than in adults when relating intake to body weight (Koch et al. 2007). Furthermore, the adverse health outcomes due to environmental chemicals may be of greater significance in children because appropriate serum levels of thyroid hormone and insulin-like growth factor I (IGF-I) are significant for growth and neurological development. In a large cohort of children, we aimed to assess the exposure to six different diphthalates by measuring their metabolites in urine samples. Furthermore, we investigated the associations with growth and serum levels of thyroid hormones and IGF-I.

## Materials and Methods

### Study participants and design

A total of 845 children 4–9 years of age were submitted to thorough clinical examinations between January 2006 and August 2007. All children had previously participated in a longitudinal cohort study, for which 1,953 women were included consecutively at their first routine obstetric control early in pregnancy at three university hospitals in Copenhagen, Denmark. Information regarding pregnancy and maternal health was obtained from medical records and questionnaires answered by the women. Gestational age of the newborn child was based on sonography, last menstrual period, and clinical evaluation of the newborn child. In case of discrepancies, sonography measurements were used. Details of the study have previously been published (Chellakooty et al. 2006). The children were examined shortly after birth and at 3, 18, and 36 months of age by standardized examinations. The length of the newborn child was measured supine with a Kiddimeter (Raven Equipment Ltd., Essex, UK) to the nearest 0.1 cm. All children participating up to 3 years of age (*n* = 1,440) were asked to participate in a follow-up study, 902 of whom consented. Of these, all children delivering a spot urine sample (*n* = 845) composed the present study population. The present study comprised measurements of height, weight, clinical assessment of pubertal stage (Tanner stage), ultrasound of the thyroid gland, including calculation of the gland volume (*n* = 839; Boas et al. 2009), blood samples (*n* = 786), and spot urine samples. In addition, the parents filled in a questionnaire on health and lifestyle.

In the present study, we present information on phthalate exposure based on data from all children from whom urine samples had been collected. For the statistical analyses of associations between phthalate concentrations and growth or endocrine measures, we excluded all children suffering from diseases prone to affect growth or endocrine status as well as all children with clinical signs of puberty. Thus, a total of 26 children were excluded because of heart disease (*n* = 3), brain tumor (*n* = 1), Langerhans cell histiocytosis (*n* = 1), diabetes (*n* = 2), epilepsy (*n* = 3), cerebral palsy (*n* = 1), chronic gastrointestinal diseases (*n* = 2), juvenile arthritis (*n* = 1), pathological thyroid function tests [two with thyroid-stimulating hormone (TSH) > 3 SD, one with T_4_ < 3 SD], or puberty (*n* = 12).

### Hormone analyses

Nonfasting peripheral venous blood samples were drawn from an antecubital vein between midmorning and late afternoon. Samples were separated by centrifugation and stored at −20°C until analyses. All analyses were carried out blinded for the technician and in random order.

TSH and thyroid hormones (T_4_, free T_4_, T_3_, and free T_3_) were measured with an electrochemiluminescence immunoassay (Modular Analytics E170; Roche GmbH, Mannheim, Germany). Total assay variations for TSH were 8.7% and 8.4% at concentrations of 0.9 and 4.9 mU/L, respectively; T_4_, 5.6% and 5.6% at 81 and 167 nmol/L; free T_4_, 6.0% and 8.1% at 12 and 30 pmol/L; T_3_, 6.7% and 6.6% at 3.2 and 6.0 nmol/L; and free T_3_, 6.4% and 6.4% at 5.3 and 15.0 pmol/L.

IGF-I and insulin-like growth factor binding protein 3 (IGFBP-3) were measured with solid-phase enzyme-labeled chemiluminescent immunometric assays (Immulite 2000; Diagnostic Products Corp., Los Angeles, CA, USA) using World Health Organization National Institute for Biological Standards and Control International Reference Reagent 87/518 and 93/560 standards, respectively. The limits of detection (LODs) were 20 and 0.1 μg/mL, respectively. Intraassay variations were < 2.1% and 4.4%, respectively; and interassay variations were < 8.9% and 5.6%, respectively.

### Urinary phthalate metabolite analyses

Spot urine samples were collected in polyethylene cups and stored as 10-mL aliquots in 20-mL glass scintillation vials with tops packed with aluminum foil at −20°C. Urine samples were analyzed for concentrations of 12 different phthalate metabolites by liquid chromatography (LC) tandem mass spectrometry with preceding enzymatic deconjugation followed by solid phase extraction. The method for preparation of samples, standard solutions, and quality controls as well as the instrumental analysis was previously described (Janjua et al. 2008) and used with the following modifications. The solvents for LC separation were as follows: A, 0.1% acetic acid in water; B, 0.1% acetic acid in acetonitrile. Solvent programming was 0.0–1.5 min, 5% B; 1.6 min, 27% B; 6.0 min, 30% B; 6.1–10.0 min, 45% B; 10.1 min, 70% B; 12.0–15.5 min, 90% B; 15.6–17.0 min, 5% B. For all analytes, the retention time on column was 6.55–13.14 min, and a good separation was obtained. The precursor and product ions (*mz*) were as previously described (Silva et al. 2007). The calibration curve range was 0.5–500 ng/mL. Method accuracy and precision were validated by repeating (*n* = 5) intraday analysis of pooled urine samples spiked with native phthalate standards (5, 10, and 50 ng/mL) and by repeating interday analysis of control urine samples spiked with low and high concentrations (*n* = 24 over a 2-month period). Mean (± SD) recovery ranged from 88% ± 8.6% to 108% ± 4.5%, and interday variation was < 10% for most analytes. LODs were calculated as previously described (Blount et al. 2000) and are listed in Table 1. All urine samples with extremely high concentrations of phthalate metabolites were reanalyzed to confirm the values.

We analyzed the following metabolites: monoethyl phthalate (MEP) from diethyl phthalate (DEP); mono-*n*-butyl phthalate and monoisobutyl phthalate (MBP; analyzed as one compound) from di-*n*-butyl and DBP; monobenzyl phthalate (MBzP) from butyl benzyl phthalate; mono-(2-ethylhexyl) phthalate (MEHP), mono(2-ethyl-5-hydroxyhexyl) phthalate (MEHHP), mono(2-ethyl-5-oxohexyl) phthalate (MEOHP), and mono(2-ethyl-5-carboxypentyl) phthalate (MECPP) from DEHP; mono-*n*-octyl phthalate (MOP) from di-*n*-octyl phthalate; and monoisononyl phthalate (MiNP) and monocarboxyisooctyl phthalate (MCiOP) from diisononyl phthalate (DiNP). In 250 randomly selected samples (125 girls, 125 boys), two additional secondary metabolites of DiNP were measured: monohydroxyisononyl phthalate (MHiNP) and monooxoisononyl phthalate (MOiNP). In samples from 100 randomly selected children, the content of both free and total (sum of free and conjugated) phthalate metabolites was determined, and the ratio was calculated (concentration of free/concentration of total phthalate metabolites).

### Urinary iodine and creatinine analyses

Because low or high serum levels of iodine are known to affect thyroid function, the overall iodine status of the population was estimated by quantifying the iodine (^127^I) concentration in 250 randomly selected urine samples using an inductively coupled plasma mass spectrometer (Agilent Technologies, Waldbron, Germany) after dilution by an aqueous solution containing tetramethylammonium hydroxide. LOD was 0.1 μg/L. Recovery of iodine (mean ±SD) was 103 ± 3% (*n* = 13). Creatinine was determined in all urine samples by colorimetric enzymatic assay.

### Statistical analyses

Statistical analyses were performed with SPSS (version 17; SPSS, Inc., Chicago, IL, USA). Body surface area (BSA) was calculated using the DuBois formula: BSA (m^2^) = 0.007184 × height (cm)^0.725^ × weight (kg)^0.425^. Standard deviation scores (SDSs) of height, weight, body mass index (BMI), and BSA were calculated based on reference material from Danish children (Andersen et al. 1982; Nysom et al. 2001). Midparental height SDS (HSDS_midpar_) was calculated as the mean height SDS of both parents. To estimate the difference between expected and observed height, we also calculated the difference between child and midparental height SDS (DiffHSDS_midpar_), the change in height SDS between 0 and 3 years (ΔHSDS_0–3_) and between 1.5 years and the current examination (ΔHSDS_childhood_).

Log transformation was applied to phthalate concentrations, phthalate ratios, TSH, IGF-I, IGFBP-3, and thyroid volume to improve the approximation of normal distribution. Statistical analyses included only phthalate metabolites measurable in more than 50% of children. For phthalate metabolite levels below the LOD, LOD divided by the square root of 2 was used. We calculated the sum of concentrations of DEHP metabolites (MEHP, MEHHP, MEOHP, and MECPP, corrected for molecular weights). To estimate associations with the combined phthalate exposure, we calculated a total phthalate score: Concentrations of each of the metabolites MEP, MBP, MBzP, MCiOP, and the sum of DEHP metabolites were divided into quartiles, and the total phthalate score was the sum of quartiles 0–3 (range, 0–15). Because evidence on dose–response relationships between phthalates and thyroid hormone levels is sparse (Hinton et al. 1986; O’Connor et al. 2002), the metabolites concerned were equally weighted in the calculation of the score.

We calculated the percentage of DEHP excreted as MEHP (MEHP%): MEHP concentration divided by the sum of all DEHP metabolite concentrations (MEHP, MEHHP, MEOHP, MECPP) × 100 (all concentrations were converted to nanomoles per milliliter). For samples with concentrations above LOD, the ratios between free and total metabolite concentrations were calculated.

We used parametric correlation analyses and the *t*-test to investigate associations between phthalate metabolite levels, age, body size, and SDS for anthropometric variables as well as sex differences. Multivariate linear regression was used to explore relationships between phthalate metabolite concentrations and serum hormone levels or growth estimates, including sex and age as covariates in analyses of hormone levels, and in addition HSDS_midpar_ and birth length in analyses of growth estimates.

Because urine was collected as spot samples, phthalate concentrations were adjusted for dilution by either dividing with the creatinine concentration or including the square root of creatinine concentrations in regression analyses. These two approaches yielded comparable estimates, so creatinine-corrected data represent phthalate divided by creatinine concentration. All statistical analyses were performed both with crude and creatinine-corrected phthalate concentrations. *p*-Values < 0.05 were considered statistically significant.

### Ethical aspects

Parents gave informed consent for the participation of their child. The study was performed according to the Helsinki II Declaration and was approved by the local ethics committee and the Danish Registry Agency.

## Results

Clinical characteristics of the 845 participating children are shown in Table 2. All urine samples contained measurable amounts of metabolites of DEP, DBP, DEHP, and DiNP. Distributions of crude and creatinine-corrected concentrations of phthalate metabolites are presented in Table 1. Crude concentrations of all phthalate metabolites were positively associated with each other, and creatinine-corrected concentrations also were positively associated with each other (*p* < 0.01 in all cases). Samples from six children contained extremely high concentrations of MBP (one boy, 6,456 μg/L), MBzP (one boy, 4,547 μg/L), DEHP metabolites (MEHHP; one girl and one boy, 1,671–1,717 μg/L), or DiNP metabolites (two boys: MCiOP, 2,063 μg/L; MHiNP, 792 μg/L). Concentrations remained high after correcting for creatinine.

Boys presented significantly higher urine concentrations of MBzP, MEHP, MEHHP, MEOHP, and MCiOP (*p* < 0.05 in all cases) than did girls. However, when corrected for creatinine, concentrations of MEP, MBP, and MECPP were higher in girls (*p* < 0.05), whereas the concentrations of MBzP, MHiNP, and MCiOP remained highest in boys (*p* < 0.05 in all cases). In correlation analyses, all phthalate metabolites were negatively associated with absolute values of height, weight, BMI, and BSA, reaching significance for MEP, MEOHP, MECPP, sum of DEHP metabolites, and total phthalate score (*r* = −0.097 to −0.074; *p* < 0.05). Only MBzP was negatively (*r* = −0.077; *p* = 0.025) correlated with age. When corrected for creatinine, all phthalate metabolites were negatively correlated with age (*r* = −0.210 to −0.106; *p* < 0.005 in all cases).

**Figure 1:**
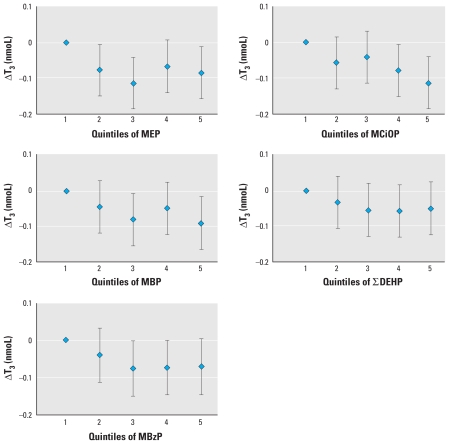
Regression coefficients (95% confidence intervals) for a change in total T_3_ (ΔT_3_) associated with quintiles of MEP, MCiOP, MBP, MBzP, and sum of DEHP metabolite concentrations (∑DEHP) (adjusted for sex and age; *n* = 758).

In the analyses of associations between phthalate concentrations and endocrine or growth measures, differences in results for boys and girls were generally within ranges consistent with random variation, but some estimates were statistically significant in only one group. In boys, phthalate metabolites were in general negatively associated with total and free T_3_, but few reached statistical significance (Table 3) [all confidence intervals are listed in Supplemental Material, Tables 1–3 (doi:10.1289/ehp.0901331)]. However, associations became nonsignificant when correcting phthalate concentrations with creatinine. We found no consistent associations with TSH, T_4_, or free T_4_ (see Supplemental Material, Tables 1 and 2). IGF-I was negatively associated with crude DEHP metabolites and MCiOP (Table 3, Figure 1), and IGFBP-3 with DEHP metabolites (*p* < 0.05). We found significantly negative associations between recent height gain (ΔHSDS_childhood_) and both crude and creatinine-corrected DEHP metabolite concentrations (*p* < 0.05) (see Supplemental Material, Table 3). All other associations between ΔHSDS_childhood_, IGF-I, IGFBP-3, and phthalate metabolites were similarly negative but not significantly. We found no consistent associations with current height (HSDS or DiffHSDS_midpar_).

In girls, all associations between peripheral thyroid hormones and unadjusted phthalate metabolites were negative, reaching significance for total T_3_ with most phthalate metabolites, for free T_3_ with MEP and DEHP metabolites (Table 3), and for total T_4_ with MEP (B = −5.19, *p* = 0.013) and total phthalate score (B = −0.48, *p* = 0.037) [Supplemental Material, Table 1 (doi:10.1289/ehp.0901331)]. IGF-I was not significantly associated with unadjusted phthalate metabolites (Table 3), but IGFBP-3 was significantly negatively associated with DEHP metabolites and MCiOP (*p* < 0.05) (see Supplemental Material, Table 2). We found significantly negative correlations between current height (HSDS or DiffHSDS_midpar_) and most crude phthalate metabolites as well as the phthalate score (see Supplemental Material, Table 3). Early height gain (ΔHSDS_0–3_) was negatively associated with MCiOP. When correcting for creatinine, only a few associations with thyroid hormones remained significant. For growth estimates, most significant associations became nonsignificant, whereas several associations with recent height gain (ΔHSDS_childhood_) became significant (see Supplemental Material, Table 3).

**Figure 2:**
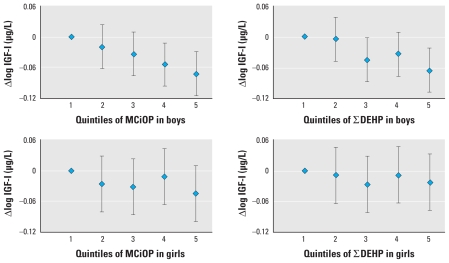
Regression coefficients (95% confidence intervals) for a change in IGF-I associated with quintiles of MCIOP and sum of DEHP metabolite (∑DEHP) concentrations in boys (*n* = 470) and girls (*n* = 297) (adjusted for age).

When analyzing boys and girls together, unadjusted phthalate metabolites showed significant negative associations with T_3_, free T_3_, and IGF-I (Figures 1 and 2, Table 3). The associations with IGF-I, but not with thyroid hormones, remained significant after correction for urinary creatinine [Supplemental Material, Figure 1 (doi:10.1289/ehp.0901331)]. Current growth measures (HSDS, DiffHSDS_midpar_ and ΔHSDS_childhood_) were significantly negatively associated with most DEHP metabolites and their sum, for both crude and creatinine-corrected concentrations (see also Supplemental Material, Table 3). Thyroid volume ranged from 1.0 to 6.8 mL and demonstrated no statistically significant sex difference. In neither boys nor girls was thyroid volume SDS associated with concentrations of phthalate metabolites (data not shown).

Mean ± SD MEHP% was 5.9 ± 2.7%. MEHP% was positively correlated with weight (*r* = 0.072, *p* = 0.039) and BSA (*r* = 0.072, *p* = 0.040) and negatively associated with T_4_ (*r* = −0.077, *p* = 0.034) and creatinine (*r* = −0.141, *p* < 0.001). Median ratios between free and total phthalate metabolite concentrations ranged from < 0.1 (MBP, MEHHP, MEOHP, MHiNP) to 0.6–0.8 (MEP, MECPP, MCiOP). Ratios correlated positively with each other (*p* ≤ 0.001 in all cases) and negatively with age (significant for MEP, MEOHP, and MHiNP) and body size.

Urinary concentration of creatinine was significantly positively associated with age, height, weight, and BSA (*p* ≤ 0.001 in all cases), and boys had higher levels of urinary creatinine than did girls (*p* = 0.001). Seventy-three urine samples were very dilute, with a creatinine concentration < 0.2 g/L. When excluding these samples from the statistical analyses, most associations between phthalate metabolites and total and free T_3_ became insignificant, although they remained negative (data not shown).

Median iodine concentration was 192.0 μg/L (282.3 μg I/g creatinine), and the 20th percentile was 115.9 μg/L. Thus, the subgroup of children tested was iodine sufficient according to World Health Organization criteria (World Health Organization et al. 2007). We repeated all the above analyses with inclusion of children with diseases or with exclusion of children born prematurely (*n* = 54) or small for gestational age (*n* = 40) and children with extremely high urinary concentrations of phthalate metabolites (*n* = 6). This did not change the overall pattern of associations. In extended versions of the regression analyses demonstrated in Table 3, we added interaction variables between phthalate metabolites and sex. In these analyses, associations between hormone levels and both phthalate levels and the interaction variable were generally nonsignificant, suggesting that estimates for boys and girls combined were valid estimates of effect and that the differences in estimates that we observed between boys and girls were generally consistent with random variation. However, we cannot rule out sex-specific effects in our study population because we had limited power to assess them. Phthalate concentrations in samples from the three excluded children with aberrant thyroid hormone concentrations were within the observed ranges in the rest of the children.

## Discussion

In this comprehensive study of phthalate exposure and health effects in children, we determined urinary excretion of 12 phthalate metabolites in 845 iodine-sufficient Danish children 4–9 years of age and related phthalate exposure measures to thyroid function and growth. The concentrations of phthalate metabolites were largely comparable to levels previously reported from other studies of children (Becker et al. 2004; Koch et al. 2007; Sathyanarayana et al. 2008; Silva et al. 2004; Wolff et al. 2007), except for relatively low levels of MEP: The geometric mean of MEP in our study group was 21 μg/L, in contrast to 91 and 177 μg/L in American studies of children (Silva et al. 2004; Teitelbaum et al. 2008). Levels of MBP were higher in Danish children than in studies from the United States (Sathyanarayana et al. 2008; Silva et al. 2004; Wolff et al. 2007) but comparable to levels in young adults in Sweden (Jonsson et al. 2005) and children in Germany (Koch et al. 2007). This may indicate regional differences in exposure.

Overall, urinary phthalate concentrations were negatively associated with thyroid hormones and IGF-I as well as with childhood growth. In some cases results were statistically significant in girls but not in boys, or vice versa. In girls, crude concentrations of phthalate metabolites were negatively associated with total and free T_3_, thus potentially reflecting an effect of phthalates on thyroid function. In boys, these associations were not as consistent, although the overall trend was also negative. Adverse effects of phthalates on peripheral thyroid hormones are supported by results from two other epidemiological studies (Huang et al. 2007; Meeker et al. 2007). Thus, a study of pregnant women found an inverse association between MBP and T_4_ and free T_4_ (Huang et al. 2007), whereas a study of adult men reported negative associations between MEHP and free T_4_ and T_3_ (Meeker et al. 2007). Evidence from animal studies is sparse, but in rats exposed to DBP (O’Connor et al. 2002) and DEHP (Hinton et al. 1986) peripheral thyroid hormones were reduced, and several studies found reduced thyroid weight and histopathology indicating thyroid hyperactivity (smaller follicles, increased number, size, and iodine content of lysosomes) after phthalate exposure (Howarth et al. 2001; Poon et al. 1997). However, in view of the very complex and multifactorial regulation of thyroid size (Hansen et al. 2004), it is not surprising that the changes in phthalate concentrations were not reflected by thyroid size alterations in our population.

Our results also revealed associations of phthalates with IGF-I in children. Few previous studies have directly addressed the effect of endocrine-disrupting chemicals on the growth hormone (GH)/IGF-I axis. Studies of IGF-I effects indicated that prenatal exposure to DBP or DEHP may lead to induction of IGF-I mRNA in reproductive tissues (Bowman et al. 2005; Lin et al. 2008), reflecting a lowering of IGF-I levels. Additionally, phthalates may potentially interact with other endocrine pathways, such as the hypothalamic–pituitary axis or androgen biosynthesis. Such complex *in vivo* effects might be expected to contribute to differences in effects according to sex. Antiandrogenic drugs may reduce IGF-I (Juul et al. 1995), and because studies have shown phthalates to have antiandrogenic properties (Gray et al. 2000), phthalates may consequently interfere with IGF-I levels. Moreover, the GH/IGF-I axis is known to stimulate the activity of peripheral deiodinases, converting T_4_ to the biologically active T_3_ (Hussain et al. 1996; Jorgensen et al. 1989). Thus, an effect on IGF-I may indirectly reduce serum levels of T_3_.

Growth rate and anthropometric measurements such as height, weight, and BSA showed overall negative associations with urinary concentrations of phthalate metabolites, consistent with an adverse effect of phthalates on growth. In support, animal studies have shown negative associations between prenatal phthalate exposure and birth weight (Tanaka 2005; Tyl et al. 2004) as well as gain of body weight (Fukuwatari et al. 2002), although conflicting data have been reported (Arcadi et al. 1998; Sharpe et al. 1995). In contrast to these studies, and to the common hypothesis of phthalates causing impaired fetal growth, Wolff et al. (2008) showed a positive association between phthalates of low molecular weight and duration of pregnancy and infant head circumference.

In our comprehensive study, we performed numerous association tests between a large number of outcome measures and 12 different phthalate metabolites. Clearly, such multiple significance testing implies a risk of obtaining “false-positive” results by chance. We therefore focused our interpretation of the results on overall trends and not on single significant associations. Thyroid hormones and growth factors are closely linked with each other and contribute significantly in the regulation of childhood growth. Thus, our observation of associations between phthalate exposure and growth supports the conclusion that phthalate exposure in this age group exerts an adverse biological effect.

However, words of caution appear necessary with regard to assessment of phthalate exposure. We collected spot urine samples, the concentrations of which will depend on recent fluid intake. Previous reports have attempted to correct, at least partly, for dilution by measuring urinary creatinine concentrations or specific gravity. However, the excretion of creatinine in children is strongly correlated with age and anthropometric variables as height, weight, and BSA (Skinner et al. 1996), as also seen in our study. In addition, there is a sex difference, with boys having higher excretion of creatinine compared with girls (Skinner et al. 1996). Thus, sex and anthropometric features per se may affect the level of the correction factor (creatinine). Consequently, when correcting phthalate concentrations by dividing by creatinine, corrected phthalate values will tend to decrease with age and body size, thus mimicking a negative association between phthalate levels and growth parameters. Such an interaction has also been demonstrated in a study of maternal urinary phthalate levels and the associations with infant outcome (Wolff et al. 2008). In contrast, associations with parameters declining with age, such as peripheral thyroid hormones in childhood, will tend to become more positive, which may be the case for the associations seen with thyroid hormones in our study.

Not only creatinine but also renal glomerular filtration rate and thus urinary volume are influenced by age, anthropometry, thyroid hormones (Iglesias and Diez 2009), and IGF-I (Feld and Hirschberg 1996). Thus, large and fast-growing children with high levels of thyroid hormones and IGF-I have a higher probability of large urinary volumes and consequently lower crude urinary concentrations of phthalate metabolites than do small children being exposed to the same amount of phthalates. Furthermore, small children may be exposed to higher levels of phthalates relative to body size (Wittassek and Angerer 2008), because small children have a higher food intake as well as a higher body surface per kilogram of body weight. Hence, the negative associations between urinary phthalate concentrations and body size or height gain may partly be explained by physiological mechanisms resulting in reverse causality. In order to adjust for the interaction between creatinine and outcome, other studies (Adibi et al. 2009) included the square root of creatinine. However, in our study, the application of this correction modus did not significantly change the statistical estimates and overall results.

Reservations should also be stated in relation to the fact that we collected only a single urine sample from each child, which may not be representative for their average exposure, although several studies concluded that a single urine sample could be moderately predictive of individual exposure over a couple of months (Hauser et al. 2004; Teitelbaum et al. 2008).

One previous study suggested that MEHP% may be a phenotypic marker of DEHP metabolism and excretion (Hauser 2008; Meeker et al. 2007). MEHP% was positively associated, and the fraction of free phthalate metabolites negatively associated, with age and anthropometric measurements, so it seems that age and body size may affect phthalate metabolism, both oxidation and glucuronidation. Other studies have found similar relations between hydrolyzed and oxidized metabolites (Becker et al. 2004). Interestingly, MEHP% was negatively associated with T_4_ in our material, which has also been reported in a previous study (Meeker et al. 2007). Thus, thyroid hormones may have an accelerating effect on the metabolism of phthalates.

## Conclusions

Our study showed negative associations between urinary phthalate concentrations and thyroid hormones, IGF-I, and growth in healthy children. Although our study was not designed to reveal the mechanism of action, the overall coherent negative associations between urine phthalate and thyroid and growth parameters may suggest causative negative roles of phthalate exposures for child health.

## Figures and Tables

**Table 1:** Sex-specific phthalate metabolite concentrations (μg/L and μg/g creatinine) in spot urine samples from 845 Danish children 4–9 years of age (examined 2006–2007).

Phthalate metabolite	LOD	Percent > LOD	Male						Female						
			Median	GM	Min	25%	75%	Max	Median	GM	Min		25%	75%	Max
Uncorrected concentration (μg/L)															
MEP	0.24	100	21	21	0.6	11	39	731	21	21	1.1		10	44	684
MBP	3.94	100	130	124	12	75	207	6,457	121	114	9.0		63	216	1,217
MBzP	1.26	86	17	2.8	0.0	6.2	37	4,548	12	0.5	0.0		3.3	31	272
MEHP	0.31	99	4.5	4.1	0.0	2.5	7.7	78	3.6	3.6	0.0		1.8	7.2	231
MEHHP	0.60	100	37	33.2	1.0	19	64	1,718	31	28	1.3		14	55	1,672
MEOHP	0.14	100	19	17	0.5	9.6	32	656	16	15	0.6		7.8	29	734
MECPP	0.43	100	30	29	1.0	16	52	676	27	27	1.7		14	49	1,755
MOP	0.04	16	0.0	0.0	0.0	0.0	0.0	11	0.0	0.0	0.0		0.0	0.0	11
MiNP	0.62	49	0.6	1.1	0.0	0.0	1.8	1,100	0.5	1.1	0.0		0.0	1.7	61
MHiNP^a^	0.31	98	6.6	5.8	0.1	3.3	11	793	4.9	4.5	0.0		2.1	8.2	400
MOiNP^a^	0.16	99	3.4	2.9	0.0	1.7	5.1	312	2.7	2.3	0.1		1.3	4.1	188
MCiOP	0.08	100	7.2	7.3	0.3	4.1	12	2,063	6.5	6.3	0.3		3.5	12	598

Creatinine-corrected concentration (μg/g creatinine)															
MEP			31	34	5.7	20	52	791	36	40	7.6	22		65	526
MBP			191	199	47.5	140	276	4,940	227	221	39	157		312	1,365
MBzP			26	25	0.0	10	49	2,916	20	22	0.0	6.9		42	474
MEHP			6.8	6.9	0.0	4.1	11	210	6.7	7.2	0.0	4.1		12	186
MEHHP			52	53	4.9	33	84	1,818	52	55	7.3	36		81	1,220
MEOHP			26	27	2.6	17	42	794	28	29	3.5	18		41	536
MECPP			43	46	6.5	29	68	1,648	49	51	8.2	33		75	1,280
MOP			0.0	0.0	0.0	0.0	0.0	10	0.0	0.0	0.0	0.0		0.0	11
MiNP			1.0	1.7	0.0	0.0	2.7	1,194	1.1	2.0	0.0	0.0		3.3	45
MHiNP^a^			8.4	8.9	1.6	5.5	14	754	7.4	7.6	0.0	4.9		9.9	292
MOiNP^a^			4.1	4.4	0.0	2.8	6.2	296	3.9	3.9	0.2	2.6		5.2	137
MCiOP			10	12	1.9	6.9	18	2,241	12	12	1.4	7.5		18	574

Abbreviations: 25%, 25th percentile; 75%, 75th percentile; GM, geometric mean; Max, maximum; Min, minimum.

a Measured in only 250 randomly selected samples.

**Table 2:** Clinical characteristics of the population [mean ± SD or *n* (%)].

Characteristic	Total (*n* = 845)	Males (*n* = 503)	Females (*n* = 342)
Age (years)	7.0 ± 1.3	6.9 ± 1.4	7.1 ± 1.1
Birth weight (kg)	3.5 ± 0.6	3.6 ± 0.6	3.5 ± 0.6
Birth length (cm)	50.8 ± 2.6	51.2 ± 2.6	50.4 ± 2.5
Gestational age (days)	278.8 ± 13.2	279.2 ± 12.6	278.3 ± 14.0
WGA (%)	−0.6 ± 12.9	−0.8 ± 12.7	−0.2 ± 13.3
Height (cm)	124.5 ± 9.5	124.4 ± 10.1	124.7 ± 8.6
Weight (kg)	24.7 ± 5.4	24.6 ± 5.6	24.9 ± 4.9
BMI (kg/m^2^)	15.8 ± 1.7	15.7 ± 1.6	15.9 ± 1.8
BSA (m^2^)	0.9 ± 0.1	0.9 ± 0.1	0.9 ± 0.1
Thyroid volume (mL)	3.1 ± 1.0	3.1 ± 1.1	3.1 ± 0.9
Urinary creatinine (g/L)	0.7 ± 0.4	0.7 ± 0.4	0.7 ± 0.4
Urinary iodine (μg/L)	216.4 ± 126.6	232.6 ± 131.5	200.4 ± 119.9
Corrected urinary iodine (μg/g creatinine)	328.7 ± 177.7	337.8 ± 178.2	319.7 ± 177.5
Maternal parity [*n* (%)]			
1	524 (62)	317 (63)	207 (61)
2	247 (29)	143 (28)	104 (31)
> 2	72 (9)	43 (9)	29 (8)
Outcome			
Singletons	803 (95)	478 (95)	325 (95)
Twins	42 (5)	25 (5)	17 (5)

WGA, weight for gestational age, given as percent deviation from expected mean weight for gestational age.

**Table 3:** Regression analyses (adjusted for age and sex) of associations between phthalate metabolites and total T_3_, free T_3_, and IGF-I in prepubertal Danish children (*n* = 758).

	Crude analysis						Creatinine-corrected analysis					
	All		Boys		Girls		All		Boys		Girls	
Outcome	B	*p*-Value	B	*p*-Value	B	*p*-Value	B	*p*-Value	B	*p*-Value	B	*p*-Value
Total T_3_												
MEP	−0.06	0.015*	−0.01	0.829	−0.12	0.001*	−0.02	0.605	0.05	0.262	−0.11	0.026*
MBP	−0.09	0.005*	−0.06	0.143	−0.14	0.007*	−0.01	0.873	−0.01	0.875	−0.02	0.780
MBzP	−0.05	0.016*	−0.04	0.150	−0.06	0.041*	−0.03	0.266	−0.03	0.412	−0.03	0.436
MCiOP	−0.07	0.017*	−0.07	0.060	−0.06	0.142	−0.01	0.837	−0.04	0.347	0.06	0.345
∑DEHP	−0.04	0.170	0.00	0.950	−0.10	0.022*	0.06	0.153	0.09	0.082	0.00	0.965
Phthalate score	−0.01	0.018*	0.00	0.518	−0.01	0.003*	0.00	0.769	0.00	0.630	0.00	0.773

Free T_3_												
MEP	−0.13	0.013*	−0.08	0.253	−0.18	0.013*	0.00	0.986	0.10	0.310	−0.14	0.179
MBP	−0.21	0.002*	−0.26	0.004*	−0.15	0.141	0.03	0.785	−0.09	0.517	0.15	0.325
MBzP	−0.08	0.032*	−0.08	0.137	−0.09	0.120	−0.02	0.710	−0.01	0.883	−0.03	0.643
MCiOP	−0.18	0.002*	−0.21	0.005*	−0.12	0.144	−0.04	0.580	−0.09	0.300	0.06	0.648
∑DEHP	−0.15	0.011*	−0.13	0.107	−0.19	0.030*	0.04	0.626	0.08	0.460	−0.05	0.689
Phthalate score	−0.01	0.006*	−0.01	0.061	−0.02	0.038*	0.00	0.562	0.00	0.844	0.00	0.662

IGF-I												
MEP	−0.01	0.213	−0.01	0.479	−0.02	0.292	−0.01	0.559	0.00	0.921	−0.02	0.309
MBP	−0.01	0.671	−0.01	0.536	0.00	0.961	0.02	0.336	0.02	0.574	0.03	0.427
MBzP	−0.01	0.383	−0.02	0.170	0.00	0.796	0.00	0.737	−0.01	0.321	0.01	0.510
MCiOP	−0.04	0.003*	−0.04	0.006*	−0.03	0.153	−0.04	0.006*	−0.05	0.020*	−0.04	0.137
∑DEHP	−0.03	0.014*	−0.05	0.002*	0.00	0.816	−0.04	0.034*	−0.07	0.003*	0.01	0.798
Phthalate score	0.00	0.027*	0.00	0.011*	0.00	0.639	0.00	0.307	0.00	0.106	0.00	0.769

Abbreviations: B, regression coefficient; ∑DEHP, sum of concentrations of all measured DEHP metabolites corrected for molecular weights.

* *p* < 0.05.
